# Utility of Coronary Artery Calcium Scores in Predicting Risk of Subclinical Cardiovascular Atherosclerotic Disease: An Analysis of Limitations to its Adoption With Policy Recommendations

**DOI:** 10.7759/cureus.14647

**Published:** 2021-04-23

**Authors:** Muhammad M Ali, Sajjad Gul, Muzna Naqvi, Laila Hakam, Asad Inayat, Sameer Saleem, Mounika Polavarpu, Mubbasher A Syed

**Affiliations:** 1 Internal Medicine, University of Toledo, Toledo, USA; 2 Internal Medicine, Order of St. Francis - St. Francis Medical Center, Peoria, USA; 3 Internal Medicine, Banner Health, Phoenix, USA; 4 Internal Medicine, Khyber Teaching Hospital, Peshawar, PAK; 5 Internal Medicine, Presence Health St Joseph Hospital, Chicago, USA; 6 Public Health, University of Toledo, Toledo, USA; 7 Interventional Cardiology, Banner Health, Pheonix, USA

**Keywords:** coronary artery calcium scans, atherosclerosis, screening

## Abstract

This survey-based analysis aims to highlight key limitations to a wider adoption of coronary artery calcium (CAC) scoring as a means of screening asymptomatic individuals for atherosclerotic cardiovascular disease. The need for a screening tool that adds objective anatomical information to historically established risk scores in the aforementioned population has been met by this imaging modality. Despite that, there has been a hesitance towards frequent usage of these scans. Within the pre-set sampling frame of the University of Toledo, a convenience sampling technique was used to reach out to 60 health care providers. The resultant responses were analyzed and discussed. In addition to identifying patients who need to be worked up further, CAC scans can also help re-stratify patients within-risk groups and inform decision-making regarding the use of lipid-lowering therapies. The public health impact of a greater but appropriate utilization of this diagnostic tool will be impactful. This analysis seeks to better understand real-life obstacles to a wider adoption of these scans and attempts to lay out policy recommendations to address these issues.

## Introduction

It is well established that cardiovascular disease is the leading cause of death in the United States and globally, with coronary artery disease (CAD) accounting for more than half of all such deaths [1]. Nearly a quarter of the patients who present with acute myocardial infarctions never experience warning signs or symptoms prior to the incident [2]. The ability to identify patients who remain asymptomatic leading up to such life-threatening events is the holy grail for researchers and physicians alike.

Cardiovascular risk scores have been historically used to fill this void, with the commonest employed tool being the Framingham Risk Score, often abbreviated as FRS [3]. A retrospective analysis by Wilson et al. in 1998 elucidated that the accuracy of FRS is 75% at best, which fails nearly a quarter of the patients [4]. The ability to characterize coronary artery calcium (CAC) by computed tomography (CT) and using that to predict the risk of future cardiovascular events has been the focus of much scientific work recently [5,6]. The presence of calcium in coronaries was historically labelled as a passive process resulting from senility, but the modern pathophysiologic understanding sees it as an active process that involves multiple mediators [6-8].

The understanding that calcification within atherosclerotic plaques of coronary arteries is a highly specific process that can be non-invasively detected and screened for led to multiple large population-based studies in the United States and European Countries as far back as 2002 [9,10]. Most databanks concurred with the initial reports of Agatston et al. published back in 1990 and furnished strong evidence of an association between detection of calcium in coronaries and future major adverse cardiovascular events in asymptomatic patients [11-13]. It is hence of high screening value to identify those patients who have a lower risk within the intermediate-risk category of asymptomatic patients based on the conventional tools, and CAC scans can fill this gap. Despite the established role and benefit of these scans as screening tools, they are scarcely used in everyday practice [14-18]. Hence, to better understand the limitations towards a wider adoption of these scans, a survey-based analysis was designed and conducted.

## Materials and methods

One of the cardinal objectives was to integrate input from health care workers who commonly participate in deciding whether to conduct CAC scoring as a screening procedure. The sampling frame was set to include providers within the faculty and academia of the University of Toledo. The aim of the survey was to extract opinions on various facets of CAC screening from all quarters including primary care physicians (PCPs), cardiology fellows, general cardiologists, and interventional cardiologists. A survey was chosen to conduct the interviews in a systematic manner to ensure that the same question was pitched with similar options to all participants to minimize bias. The need to conduct the analysis in a systematic manner, as well as the increasing concerns with in-person interactions during the COVID 19 pandemic, culminated in the decision to use a web-based online questioning tool to request opinions from participating health care workers. A well-known survey designing website called surveymonkey.com was utilized to design a simple questionnaire comprising eight close-ended questions. The questions were pointed, touched upon most aspects being investigated, and were brief and few to ensure that filling the survey takes under 3 minutes to complete. Within the pre-set sampling frame of the University of Toledo, a convenience sampling technique was used to reach out to 60 health care providers including 25 primary care and family practice physicians, 15 general cardiology practitioners (including three nurse practitioners that work closely with them), 10 interventional cardiologists, and 10 cardiology fellows in training (nine general cardiology and one interventional cardiology fellow). It can also be argued that the final sample was a volunteer sample since respondents volunteered to respond. All invitees were emailed requesting them to fill out the survey and explaining the subject and brevity of the questionnaire. The resultant responses were arranged into tables and figures using the online analytic tools on the web-based survey engine.

## Results

**Figure 1:**
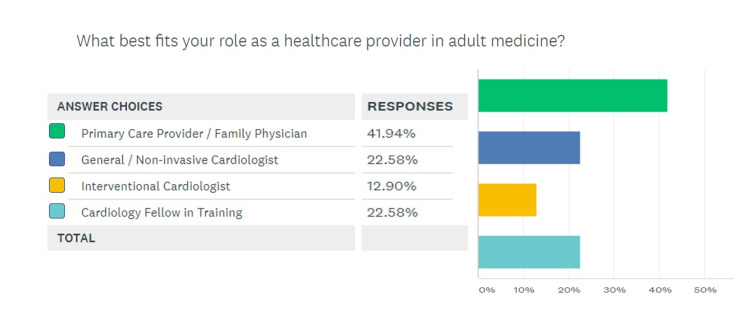
Identifying survey respondents according to their role to better delineate the sample

**Figure 2:**
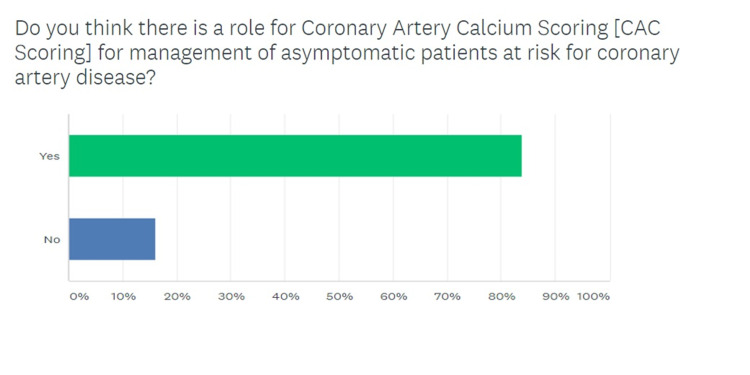
Querying the role of CAC scoring as a screening tool for coronary atherosclerosis in asymptomatic patients CAC, coronary artery calcium

**Figure 3:**
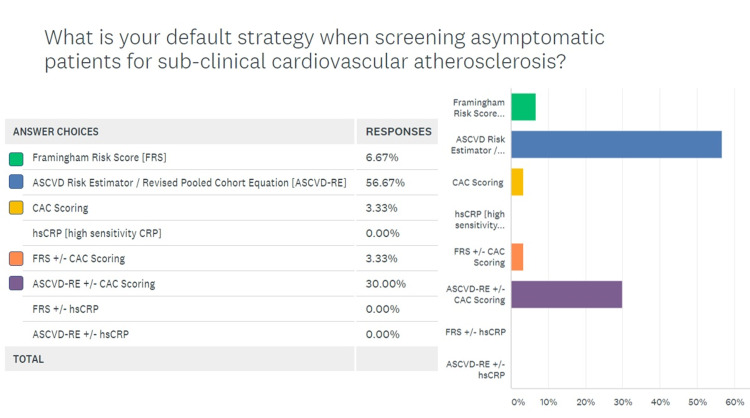
Surveying the default strategy for screening asymptomatic individuals for coronary atherosclerosis

A response rate of 50% (30 responses for 60 invitations) was generated by the survey. The breakdown for various categories of health care providers responding to the survey is given in Figure 1, which shows that nearly half the respondents were PCPs. Figure 2 demonstrates that 86.6% (26/30) respondents agreed that CAC scans had a role to play in the management of asymptomatic patients with a risk for CAD. Figure 3 shows the response to the third question in the survey, which queried the default strategy used by these providers to screen asymptomatic patients for coronary disease. While 56.6% providers chose ASCVD-RE (Atherosclerotic Cardiovascular Disease Risk Estimator) alone, another 30% of respondents reported relying on a combination of the aforementioned risk estimator and CAC screening.

**Figure 4:**
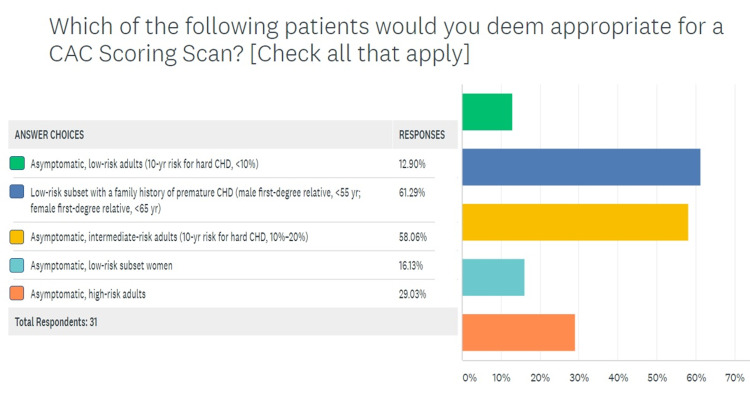
The challenge of appropriate patient selection for ordering CAC scans CAC, coronary artery calcium

**Figure 5:**
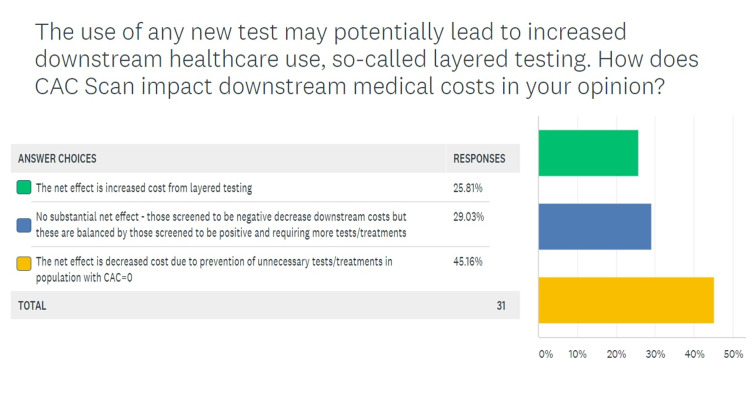
Opinions on downstream testing with CAC scans CAC, coronary artery calcium

In the fourth question, respondents were asked to choose appropriate scenarios to order a CAC scan for atherosclerotic screening. Figure 4 shows that most providers agreed that CAC scores may add value to the evaluation in low-risk subsets with a family history of premature CAD (63.3% of providers) and intermediate-risk subsets that have a 10-20% risk of CAD based on risk factor estimations (56.6% of the providers). Only 16.6% agreed with screening low-risk women, while 30% of providers deemed it appropriate to screen high-risk asymptomatic patients. In response to the fifth question on the survey, most respondents (46.6%) agreed that downstream testing costs are decreased if CAC scans are performed appropriately (Figure 5).

**Figure 6:**
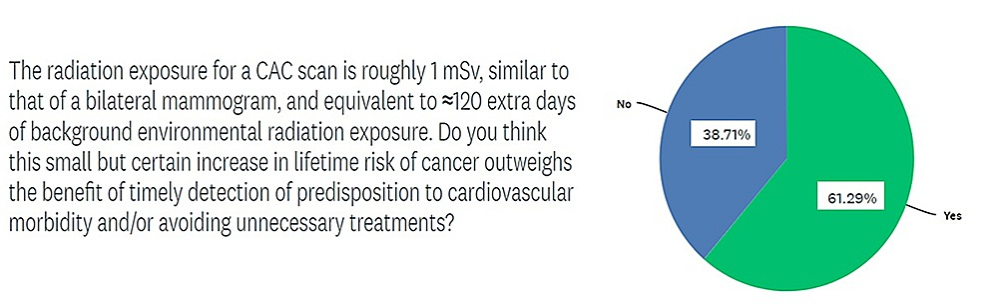
Survey respondents on the risk of radiation exposure with CAC scans CAC, coronary artery calcium

**Figure 7:**
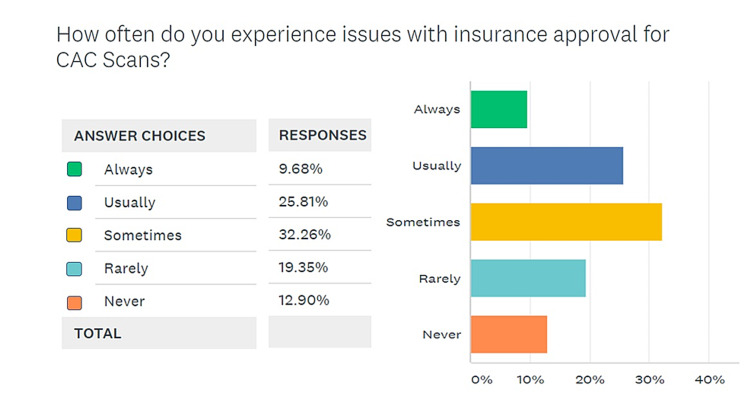
The frequency of challenges from insurance companies regarding approval of CAC scans CAC, coronary artery calcium

**Figure 8:**
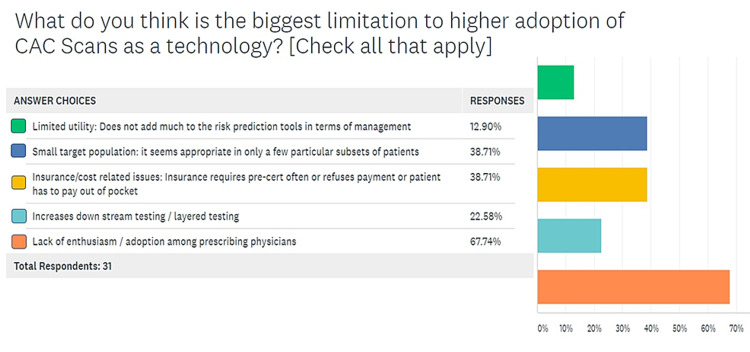
Stratifying the limitations to a wider adoption of CAC scans as a screening tool CAC, coronary artery calcium

When asked if benefit of an indicated CAC scan outweighed a 1-mSv exposure to radiation and the associated carcinogenic potential, in the sixth question on the survey, a larger number of providers believed that the benefit was enough to outweigh the risk, but 40% (12 of 30) respondents felt otherwise, as shown in Figure 6. The seventh question asked respondents about the frequency with which they faced issues with insurance approval for CAC scoring scans. Only three of 30 respondents reported to have “never” encountered such problems, while 70% of respondents answered either “always”, “usually”, or “sometimes” (Figure 7). In the same vein, the last and eighth question on the survey was meant to investigate the issues perceived as major obstacles to a greater adoption of CAC scans as a screening modality among the providers responding to the survey. Of the respondents, 70% agreed that lack of enthusiasm/adoption was the biggest obstacle to higher usage followed by a small target population (40% respondents) and insurance/cost-related issues for 38.3% respondents. These findings are summarized in Figure 8.

## Discussion

After the detailed assessment of the scientific rationale of using coronary calcium scoring modalities as a screening test for asymptomatic patients with either intermediate-risk profile or a family history of premature CAD, and its utility in these subsets, the survey results above can help identify a pattern of modifiable obstacles that need to be rectified.

The survey was able to elicit response from many stakeholders in the CAC scanning paradigm. This was partly due to the simple and concise design of the survey in accordance with the findings of Liu and Wronski that assert that long and detailed surveys rarely achieve a good response rate [19]. Most respondents were PCPs since 25 out of 60 surveys were sent out to that group of providers, which have a central role in screening asymptomatic patients. Nearly 87% of respondents agreed that CAC scans had a role in risk management for asymptomatic patients, but a majority (56.6%) chose the ASCVD-RE alone, a risk estimation tool that is rooted in risk factor analysis, over a combination of ASCVD-RE and CAC scan (30%) [20]. Evidence suggests that this approach is less accurate [17]. Similarly, the response to question 4 (Figure 4) shows that even in providers that are routinely seeing adult patients in whom a CAC scan maybe indicated, there is a lot of intellectual dissonance. While multiple studies including the landmark publication by Lakoski et al. have validated the indication to screen low-risk women given the atypical symptomology for coronary disease in this gender, only 16.6% agreed with such a strategy [21]. Moreover, a staggering 30% of providers deemed it appropriate to screen high-risk asymptomatic patients, which has been shown to be inefficient based on the Bayesian theorem [22].

Another common theme surrounding the academic discussion on increased adoption of CAC scanning has been the financial and radiological costs of this screening tool. It was initially proposed that routinely conducting CAC scans in asymptomatic adults would increase downstream testing and hence overall cost. Some preliminary studies that were critiqued for inappropriate patient selection suggested an unnecessary increase in downstream testing costs [23]. A randomized trial by Rozanski et al. in 2011 demonstrated that compared to no scanning, CAC scanning was associated with much better coronary disease risk factor control without increasing downstream medical testing [24]. Similarly, another commonly cited risk associated with routine use of CAC screening is the radiation exposure associated with it. Budoff et al. established that electron beam CT (EBCT) has an effective radiation dose of 0.7 to 1.0 mSv in men and 0.9 to 1.3 mSv in women [5]. Because of its slightly longer exposure time, multidetector CT (MDCT) has a slightly higher effective radiation dose of 1.0 to 1.5 mSv in men and 1.1 to 1.9 mSv in women. Our respondents were asked if the benefit of an indicated CAC scan outweighed a 1-mSv exposure to radiation and the associated carcinogenic potential. While 60% believed that the benefit was enough to outweigh the risk, 40% (12 of 30) respondents felt otherwise. Such a discernible split on a key question regarding the risk-benefit ratio on CAC scans will surely impact the adoption of this technology. Annual background radiation exposure from natural sources usually ranges from 1.5 to 3.5 mSv and can go up to 50 mSv. Moreover, using the relative risk reduction with 20 mg of rosuvastatin, the five-year number needed to treat (NNT) to prevent one cardiovascular event varies from 124 for those with a CAC score of 0 to 19 for those with a CACS above 100. Hence, identifying 19 patients with a CAC score above 100 and risk stratifying them to a more targeted therapy can prevent a major cardiovascular event at the cost of one-time exposure to 1 mSv of radiation.

One of the major obstacles to greater adoption of CAC scores as a screening modality pertains to issues with pre-approval from insurance agencies. A typical CAC screening costs 500 dollars, with MDCT being generally less expensive than EBCT [25]. If insurance refuses coverage or asks for a hectic pre-approval process, physicians are likely to get discouraged and avoid a modality. Other options are to ask patients to pay out-of-pocket, which again adds more workload to a provider and more cost to the patient. Figure 7 shows that only 10% of respondents reported to have “never” encountered such problems, while a staggering 70% of respondents to have encountered issues with insurance with varying frequencies. Issues with insurance approval were hence identified as a major obstacle in higher adoption of this modality.

In the last question on the survey, which was meant to investigate the issues perceived as major obstacles to a greater adoption of CAC scans, 70% of respondents reported that lack of enthusiasm/adoption among providers was the biggest obstacle to higher usage (Figure 8). In face of such an evident divide within end-users and providers within a singular healthcare system, on every aspect of the test, from its indications to its financial and radiation costs, it is not hard to imagine the lack of enthusiasm surrounding its adoption. Moreover, it is a fact that the niche populations that derive maximum benefits from the screen are small subsets that need to be appropriately identified for the sake of patient benefit as well as insurance approval. When intellectual dissonance on a subject is compounded by approval problems from insurance agencies, it is only natural that most providers would be inclined to use simpler risk factor based tools alone to arrive at their decisions.

Recommendations

1. For trainees who are still undergoing training in internal medicine and cardiology, there should be a seminar or workshop geared to better equip these future practitioners towards the usage of this useful tool. For those who are currently in practice, educational online modules and training workshops with CME (Continuing Medical Education) credits can be offered to help refresh salient applications of CAC scanning. Moreover, primary care offices can be provided with posters of simplified decision-making algorithms and charts to facilitate appropriate ordering.

2. A rectification of misconceptions regarding cost-effectiveness and radiation exposure can be achieved by embedding modules on downstream testing as well as risk-benefit ratios in terms of radiation exposure within the aforementioned training workshops and online modules.

3. To address the difficulty with insurance pre-approval for these scans, it would be productive to reach out to physicians that have had higher success in achieving approval for their ordered CAC scan and recruiting them to offer insights during workshops and seminars to their colleagues. Such pioneers can also lead quality improvement initiatives for ancillary staff in outpatient offices where such tests are often ordered. If improved patient selection by providers is accompanied by appropriate documentation by ancillary staff, the resultant improved approval from insurance can itself be a good stimulus for increased adoption of this scan.

4. The biggest limitation to higher adoption of CAC scans as a screening tool seems to be a lack of enthusiasm among providers. The best method to stimulate enthusiasm among physicians is to show results. Compiling a system-wide registry of all CAC scans conducted and resultant re-stratification can generate more interest among providers.

## Conclusions

CAC scoring is a viable coronary atherosclerotic screening modality that can be used to great effect in asymptomatic adults if ordering physicians are well-trained in patient selection. In addition to identifying patients who need to be worked up further, it can also help re-stratify patients within-risk groups and inform decision-making regarding the use of lipid-lowering therapies. Many of the obstacles in adoption of this screening modality can be overcome by focused training modules, educational seminars, and appropriate documentation. A concerted quality improvement initiative can achieve dramatic improvements in appropriate adoption of this technology.
